# A YAP/TAZ-miR-130/301 molecular circuit exerts systems-level control of fibrosis in a network of human diseases and physiologic conditions

**DOI:** 10.1038/srep18277

**Published:** 2015-12-15

**Authors:** Thomas Bertero, Katherine A. Cottrill, Sofia Annis, Balkrishen Bhat, Bernadette R. Gochuico, Juan C. Osorio, Ivan Rosas, Kathleen J. Haley, Kathleen E. Corey, Raymond T. Chung, B. Nelson Chau, Stephen Y. Chan

**Affiliations:** 1 Divisions of Cardiovascular and Network Medicine, Brigham and Women’s Hospital, Harvard Medical School, Boston, MA USA; 2 Regulus Therapeutics, San Diego, CA, USA; 3 National Institutes of Health, Bethesda, MD, USA; 4 Division of Pulmonary and Critical Care Medicine, Brigham and Women’s Hospital, Boston, MA, USA; 5 Liver Center and Gastrointestinal Division, Massachusetts General Hospital, Boston, MA, USA

## Abstract

The molecular origins of fibrosis affecting multiple tissue beds remain incompletely defined. Previously, we delineated the critical role of the control of extracellular matrix (ECM) stiffening by the mechanosensitive microRNA-130/301 family, as activated by the YAP/TAZ co-transcription factors, in promoting pulmonary hypertension (PH). We hypothesized that similar mechanisms may dictate fibrosis in other tissue beds beyond the pulmonary vasculature. Employing an *in silico* combination of microRNA target prediction, transcriptomic analysis of 137 human diseases and physiologic states, and advanced gene network modeling, we predicted the microRNA-130/301 family as a master regulator of fibrotic pathways across a cohort of seemingly disparate diseases and conditions. In two such diseases (pulmonary fibrosis and liver fibrosis), inhibition of microRNA-130/301 prevented the induction of ECM modification, YAP/TAZ, and downstream tissue fibrosis. Thus, mechanical forces act through a central feedback circuit between microRNA-130/301 and YAP/TAZ to sustain a common fibrotic phenotype across a network of human physiologic and pathophysiologic states. Such re-conceptualization of interconnections based on shared systems of disease and non-disease gene networks may have broad implications for future convergent diagnostic and therapeutic strategies.

Increases in tissue stiffness and intracellular tension are controlled by molecular changes in the extracellular matrix (ECM) and are common features of fibrosis, as found in health and disease^1^,^2^. ECM remodeling is a complex process, occurring through changes in the balance between matrix deposition and matrix degradation and through collagen crosslinking enzymes such as lysyl oxidase (LOX). Two related transcriptional coactivators, YAP (Yes Associated Protein 1) and TAZ (or WWRT1) are crucial for mechanotransduction, a process that converts extracellular mechanical cues into intracellular signaling^3^ and regulates cellular proliferation and survival^4^,^5^ as well as organ growth^6^. These coactivators have been increasingly appreciated as active factors that control ECM plasticity^7^ in normal development and physiology as well as pathologic fibrosis^8^. Recently, we described a critical role of the control of ECM stiffening by the mechanosensitive microRNA-130/301 family, as activated by YAP/TAZ, in promoting pulmonary hypertension (PH)^9^. Identification of such a self-amplifying feedback loop in PH leading to perivascular fibrosis suggested that similar molecular mechanisms involving extracellular biomechanical cues and microRNA (miRNA) activity play important roles in other fibrotic contexts.

More specifically, individual miRNA families often control multiple target genes and phenotypes, making them attractive candidates as upstream “master” regulators of seemingly diverse processes, including cell-cell and cell-matrix interactions^10^. Crosstalk between miRNA biology and the biomechanical ECM properties across tissue types, however, has been largely unexplored. Recently, we have utilized network theory to predict *in silico* those dominant miRNAs governing a specific disease gene network^11^,^12^. Given the broad scope of human conditions where ECM plasticity may figure prominently, we hypothesized that remodeling of the ECM could be a pathogenic or normal physiologic feature shared among seemingly disparate conditions, and miRNAs could be an upstream regulator of this molecular cascade. In order to address these questions we developed an advanced network-based approach to search for a global miRNA regulator(s) of human fibrotic phenotypes across 137 different human diseases and physiologic conditions.

In doing so, we have now identified miR-130/301 as a master regulator of ECM biology across a cohort of physiologic and pathophysiologic states – all related by a shared signature of fibrosis-relevant genes. Overall, these results define this miRNA family as a crucial point of communication between biomechanical stress and fibrosis in a network of *in vivo* contexts. These findings also emphasize the increasingly attractive candidacy of miR-130/301 for selective therapeutic targeting in such related diseases.

## Results

### Network analysis reveals that miR-130/301 members target a shared cohort of fibrotic genes across human diseases and physiologic states related to PH

**Figure 1:**
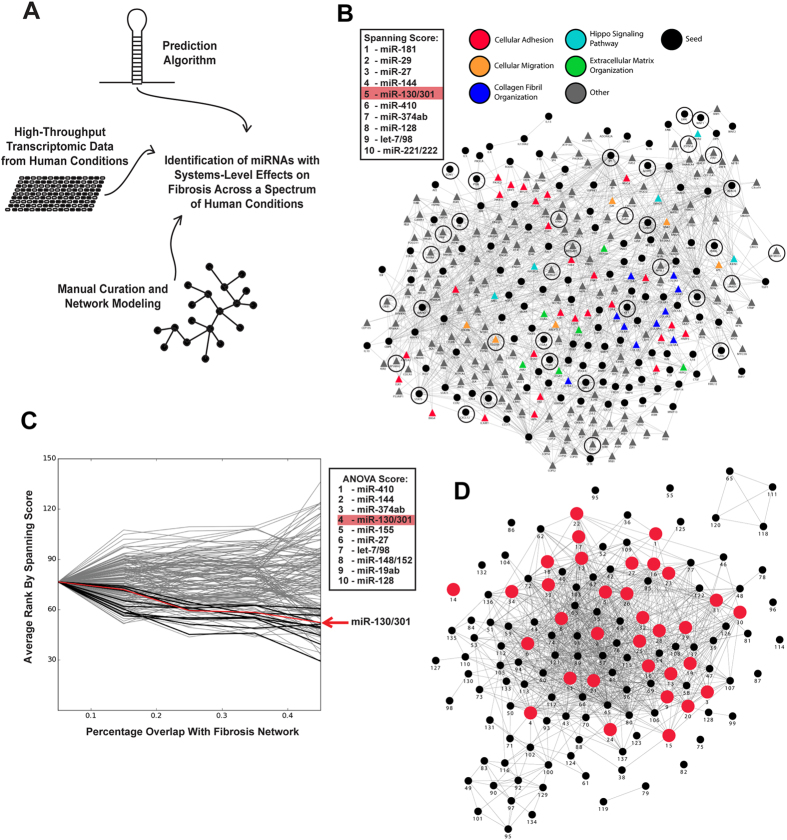
miR-130/301-specific fibrotic activity is active throughout a network of human diseases and physiologic conditions. (**A**) Strategy used to identify miRNAs that exert systems-level control over fibrosis. (**B**) A fibrosis network, composed of known fibrotic genes (seed genes, circles) and their closest first-degree interactors (triangles, see Supplemental). Color-coding denotes inclusion in known annotated pathways relevant to fibrosis and the ECM (from the GO, Kegg, Reactome, NCBI PID, and Biocarta databases), and demonstrates the relevance of incorporated first-degree interactors to fibrotic processes. miR-130/301 was ranked among the top five miRNAs by spanning score in this network (targets encircled in black), reflecting its robust, systems-level control over fibrosis and matrix remodeling. (**C**) Based on transcriptomic profiling and network construction (see Methods), 137 networks representing various disease and physiologic states were grouped into cohorts, according to overlap with the fibrosis network. MiRNAs were then scored by one-way inverse correlation (one-way ANOVA) of their average assigned spanning score rank across cohorts. miR-130/301 was ranked among the top five miRNAs, underlining its importance to diseases with a strong fibrotic component (red box). (**D**) An abbreviated network was generated from 137 diseases and physiologic states (indexed in Table S1). Edges among conditions denote significant overlap (hypergeometric p-value < 0.1). Conditions in red were ranked highly based on their interconnectedness with the fibrosis network and the miR-130/301 family (top 25%, as ranked in Table S1), and all were found to share a distinct cohort of fibrotic genes embedded in the overlap with the PH network. The prevalence of fibrotic states predicted to involve miR-130/301 provides evidence for a shared fibrotic program controlled by this miRNA family in a wide range of human pathology and conditions beyond the pulmonary vasculature (see Table S3).

Given the importance of the YAP/TAZ-miR-130/301 circuit in PH^9^, we postulated that this feedback loop may be similarly active in controlling ECM plasticity in other fibrotic states. To define miRNAs that carry overarching regulatory control of tissue fibrosis across different contexts, we employed an *in silico* combination of miRNA target prediction, transcriptomic analysis across 137 human disease and physiologic states, and advanced gene network modeling (Fig. 1A). First, to identify miRNAs with global regulatory effects in a given condition, we previously described a “miRNA spanning score” (see **Methods**) based on the number and architectural distribution of predicted miRNA targets within a representative gene network^12^. Utilizing an *in silico* “fibrosis network”^9^ composed of curated seed genes known to be *causatively* involved in ECM remodeling and their first degree interactors (Fig. 1B), we found a broad and diverse contingent of factors related to ECM remodeling within the predicted pool of miR-130/301 target genes and their related network neighbors.

To determine the relationship of these ECM-specific miRNA actions to other physiologic contexts, we compiled a suite of transcriptomic datasets of affected human tissue as compared with normal controls (n = 137) from publically available databases, representing a wide cross-section of human pathology and physiologic states (see **Methods** and Table S1). To control for statistical noise from wide variations in network size, specific disease gene networks were constructed by selecting the top 250 (by fold-change) significantly differentially expressed genes in each array, compared with non-affected controls. We cross-referenced these seed genes with the consolidated interactome and incorporated well-connected first-degree interactors, in order to “amplify” the molecular signal of these putative systems-level changes. Each network was categorized into one of four cohorts, as defined by increasing overlap with the fibrosis network. For each of the 137 networks, miRNAs were then ranked by spanning score, as described above. Although it appeared that many miRNAs may have relevant actions in fibrosis, with these data, we identified miR-130/301 family members among the most highly ranked miRNAs (Rank #4) with a robust one-way inverse correlation (one way ANOVA) between their assigned spanning score rank and the size of the fibrotic component for each of the 137 networks (Fig. 1C, Table S2). Coupled with the top ranking of this miRNA family by spanning score in relation to the fibrosis network directly (Fig. 1B), these findings predicted that miR-130/301 members are integral to the fibrotic program across a variety of diseases and tissue beds. Moreover, consistent with the known relevance of miR-130/301 in PH^12^, among the 137 networks described above, a subset, ranked highly by their interconnectedness with the fibrosis network and the miR-130/301 family (Table S1, S3), was found to share a distinct cohort of fibrotic genes embedded in the overlap with a previously reported^12^ PH disease gene network (Fig. 1D, Table S3). Thus, a combination of network analyses predicted a unique position for the miR-130/301 family at the intersection of fibrotic gene programming and this set of associated diseases and physiologic states.

### miR-130/301 expression correlates with activation of YAP/TAZ, the PPARY-APOE-LRP8 axis, and matrix stiffening in pulmonary fibrosis and liver fibrosis

**Figure 2:**
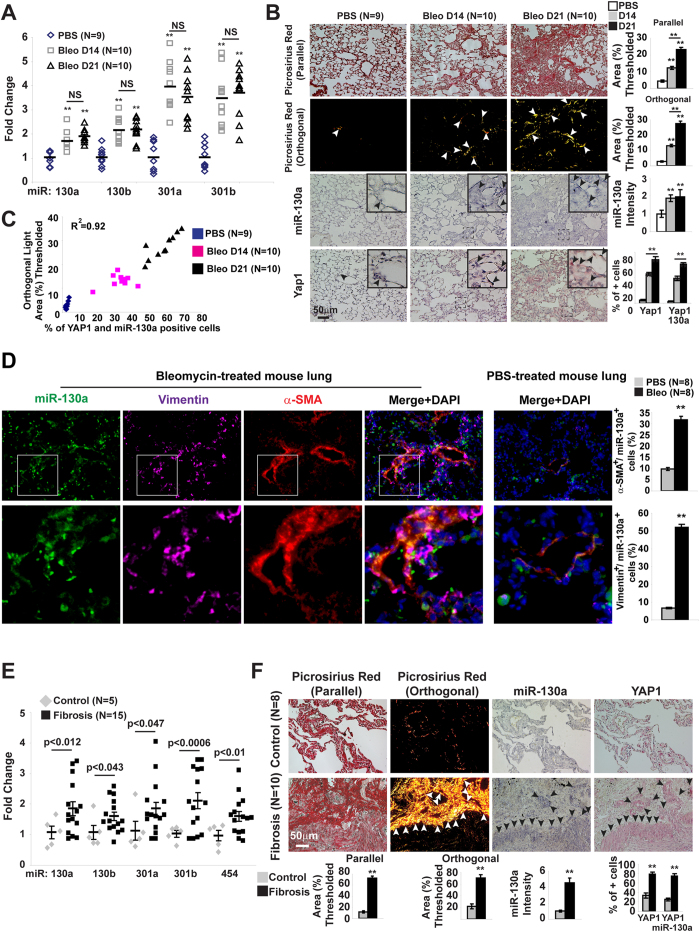
Positive correlation of ECM stiffening and YAP/TAZ-dependent expression of miR-130/301 in mice and humans suffering from lung fibrosis. A mouse model of lung fibrosis (bleomycin-induction, n = 9–10/group) was analyzed. miR-130/301 was significantly increased by RT-qPCR (**A**), and serial sections of lung (**B**) displayed increased collagen (Picrosirius Red), miR-130a, and YAP by *in situ* hybridization. (**C**) In diseased lung, miR-130a and YAP nuclear localization was positively correlated with collagen crosslinking. (**D**) *In situ* staining for miR-130a and fibroblast markers (vimentin and α–SMA) was performed by fluorescent microscopy. Vimentin/miR-130a and α–SMA/miR-130a positive cells were increased in bleomycin-treated lung tissue (n = 8 per groups; 5 20X fields per slide were quantified). (**E,F**) In the lungs of patients with idiopathic pulmonary fibrosis, miR-130/301 and YAP1 were increased by RT-qPCR (**E**; [controls n = 5, fibrosis n = 15]) and *in situ* stain (**F**; [controls n = 8, fibrosis n = 10]). (See also Fig.S1). Data are expressed as mean ± SEM (*P < 0.05; ** P < 0.01).

To demonstrate the putative unifying biology of miR-130/301 in this cohort of physiologic states, pulmonary fibrosis (Index Diseases #11 and #19, Table S1) and fibrotic liver disease (Index Disease #4, Table S1) were selected for further interrogation. In mouse models of bleomycin-induced pulmonary fibrosis (Fig. 2), miR-130/301 expression was increased (Fig. 2A,B). Consistent with induction of miR-130/301 by the mechanosensitive YAP/TAZ transcription factors in PH^9^, a positive correlation was observed among miR-130a expression, YAP nuclear localization, and downstream collagen crosslinking in pulmonary fibrosis (Fig. 2C). Furthermore, consistent with the direct down-regulation of the associated factors peroxisome proliferator-activated receptor gamma (PPARγ), apolipoprotein E (APOE) and the apolipoprotein E receptor LRP8 by miR-130/301 in PH 9, both Pparγ and Lrp8 were decreased in pulmonary fibrosis (**Fig.S1**). To define the exact cell types in which miR-130/301 was up-regulated, an *in situ* protocol was developed to stain simultaneously for miRNA and protein (see **Methods**). In the lung of mice treated with bleomycin, miR-130a expression was up-regulated in parenchymal fibroblasts, as demonstrated by co-localization of miR-130a with vimentin, a fibroblast marker, and α-smooth muscle actin (α–SMA), a marker of both activated fibroblasts and smooth muscle cells (Fig. 2D). Correspondingly, as predicted by our network algorithm (Index Diseases #11 and #19, Table S1), the same relationships between miR-130/301, YAP/TAZ, and collagen crosslinking were observed in pulmonary tissue derived from a cohort of patients suffering from idiopathic pulmonary fibrosis (Fig. 2E,F, Fig. S1). Thus, beyond PH, the YAP/TAZ-miR-130/301 feedback loop is active in pulmonary fibrosis and specifically in fibroblasts anatomically far removed from the pulmonary vasculature itself.

**Figure 3:**
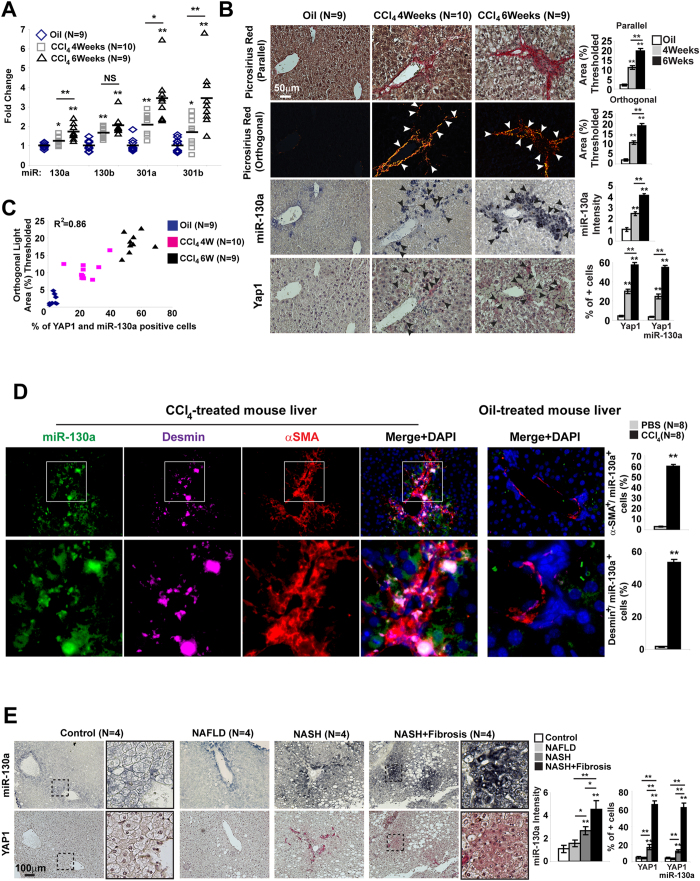
Positive correlation of ECM stiffening and YAP/TAZ-dependent expression of miR-130/301 in mice and humans suffering from liver fibrosis. A mouse model of liver fibrosis (CCl_4_-induction, n = 9–10/group) was analyzed. miR-130/301 was significantly increased by RT-qPCR (**A**), and serial sections of lung (**B**) displayed increased collagen (Picrosirius Red), miR-130a, and YAP by *in situ* hybridization. (**C**) In diseased lung, miR-130a and YAP nuclear localization was positively correlated with collagen crosslinking. (**D**) *In situ* staining for miR-130a miR-130a and stellate cell markers (desmin and α–SMA) was performed by fluorescent microscopy. Desmin/miR-130a and a–SMA/miR-130a positive cells were increased in CCl_4_-treated liver (n = 8 per groups; 5 20X fields per slide were quantified). (**E**) Similarly, *in situ* stain demonstrated an increase in miR-130a and YAP in fibrotic liver tissue of patients (n = 4 per group) with non-alcoholic steatohepatitis (NASH+fibrosis) (see also Table S4 and Fig.S2). Data are expressed as mean ± SEM (*P < 0.05; **P < 0.01).

Similarly, in a mouse model of carbon tetrachloride (CCl_4_)-induced liver fibrosis (Fig.3), miR-130/301 was increased (Fig. 3A,B) and Pparγ and Lrp8 were correspondingly decreased (**Fig.S2**), accompanied by a positive correlation among collagen crosslinking, miR-130a expression, and YAP nuclear localization (Fig. 3C). Via *in situ* liver stain of mice treated with CCl_4_, miR-130a expression co-localized with both desmin, a stellate cell marker, and α–SMA (Fig. 3D). Similarly, as guided by our network predictions regarding various fibrotic liver diseases (for instance, Index Disease #4, Table S1), miR-130a and YAP were found to be increased in fibrotic human liver tissue – in this case stemming from nonalcoholic steatohepatitis (Fig. 3E patient demographics in Table S4). Thus, as delineated by our *in silico* predictions, distinct from PH, the YAP/TAZ-miR-130/301 circuit is activated across both pulmonary and hepatic diseases in animals and humans.

### Forced miR-130/301 expression activates ECM remodeling and liver fibrosis in mice

**Figure 4:**
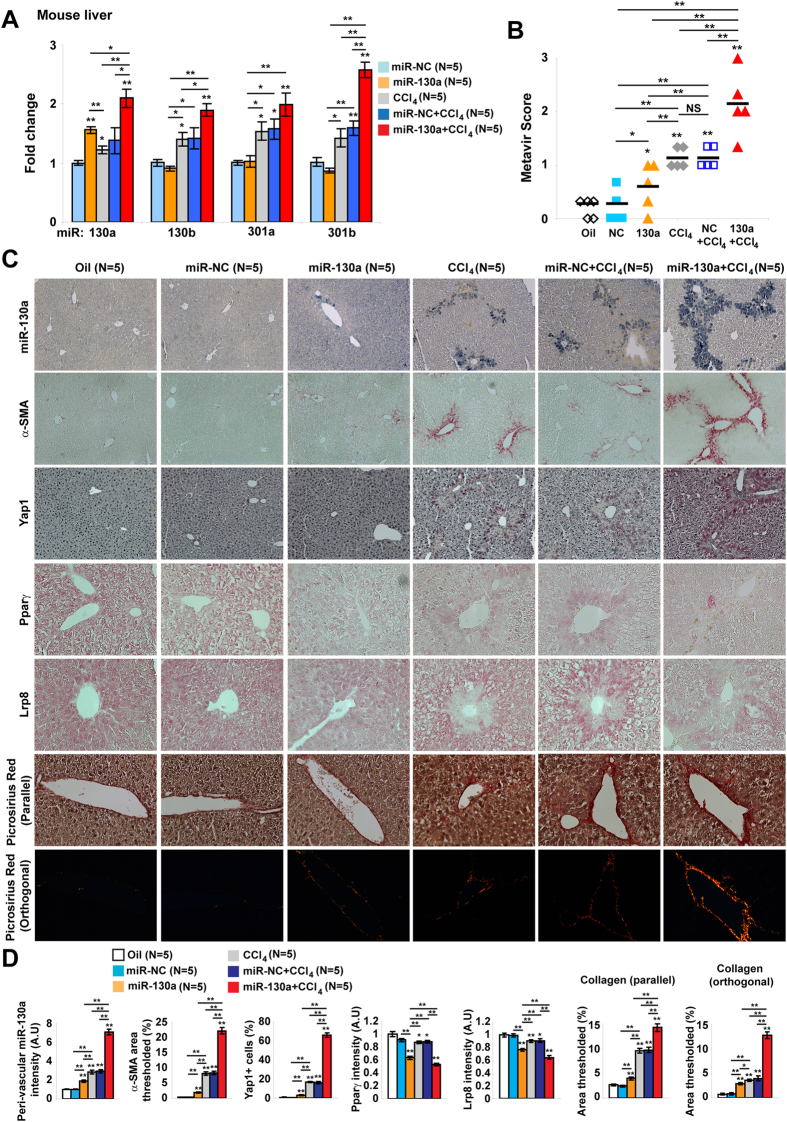
miR-130/301 overexpression activates ECM remodeling and liver fibrosis progression in mouse. (**A**) As assessed by RT-qPCR, serial intraperitoneal delivery of miR-130a mimic oligonucleotide (miR-130a) increased miR-130a in whole liver in mice as compared with control (miR-NC), either with or without weekly injection of suboptimal CCl_4_ dose. (**B**) By Metavir score, either a suboptimal dose of CCl_4_ or miR-130a independently increased liver fibrosis, while miR-130a+CCl_4_ even more robustly increased such fibrosis. (**C**,**D)** *In situ* hybridization of miR-130a (top row) confirmed effective delivery in the liver of mice. Immunohistochemistry also revealed that miR-130a decreased Lrp8 and Pparγ as well as slightly increased YAP nuclear localization and collagen deposition and crosslinking. Moreover, miR-130a+CCl_4_ more robustly decreased Lrp8 and Pparγ as well as more robustly increased YAP nuclear localization, collagen deposition, and crosslinking.

We previously demonstrated that forced expression of miR-130/301 in the lung of mice is sufficient to induce pulmonary hypertension^12^ and pulmonary vessel fibrosis^9^. To determine if miR-130a is sufficient to induce liver fibrosis, chronic liver expression of miR-130a was studied. MiRNA delivery was achieved by serial (every 3 days during 4 weeks) intraperitoneal injections of liposomally encapsulated miR-130a oligonucleotide mimics in presence or absence of suboptimal dose of CCl_4_ (0.1 mL per kg body by week). This protocol led to up-regulated miR-130a expression (but not other family members) in whole liver tissue (Fig. 4A). Such delivery down-regulated target gene expression of Pparγ and Lrp8 as well as modestly activated Yap1 nuclear localization and collagen crosslinking (Fig. 4B–D). These effects were enhanced by delivery of both miR-130a along with a decreased dose of CCl_4_, promoting a robust down-regulation of Lrp8 and Pparγ and more substantially increased Yap1 activation and collagen crosslinking, compared to CCl_4_ alone, CCl_4_+miR-control (miR-NC), or miR-130a alone (Fig. 4C,D). Moreover, forced miR-130a and increased ECM stiffening was sufficient to activate a self-amplifying feedback loop and further increase endogenous miR-130/301 family expression (Fig.4A). Taken together, consistent with the miR-130a-dependent actions in the pulmonary vascular space, these results demonstrated that this miRNA is sufficient to induce liver fibrosis as well.

### Inhibition of the miR-130/301 family prevents ECM remodeling and disease progression in mouse models of pulmonary fibrosis and liver fibrosis

**Figure 5:**
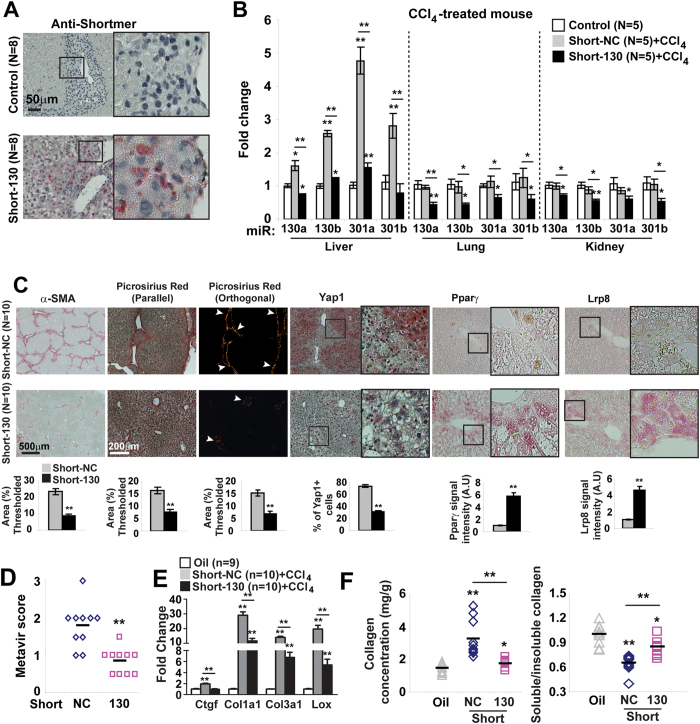
miR-130/301 inhibition ameliorates liver fibrosis. In a model of liver fibrosis (CCl_4_-induction), mice were treated with Short-NC or Short-130 (n = 8/10 per group). Short-130 delivery (n = 8 per group; **A**) and activity (n = 5 per group; (**B**) were confirmed. Short-130 decreased fibrosis, as assessed by α–SMA and collagen staining (**C**), as well as Metavir score (**D**). By *in situ* stain (**C**), Short-130 decreased vascular YAP nuclear localization and increased Pparγ and Lrp8. Short-130 also decreased transcript expression of Ctgf, collagen isoforms, and Lox (**E**) as well as decreased collagen deposition and collagen crosslinking (**F**). Data are expressed as mean ± SEM (*P < 0.05; **P < 0.01).

**Figure 6:**
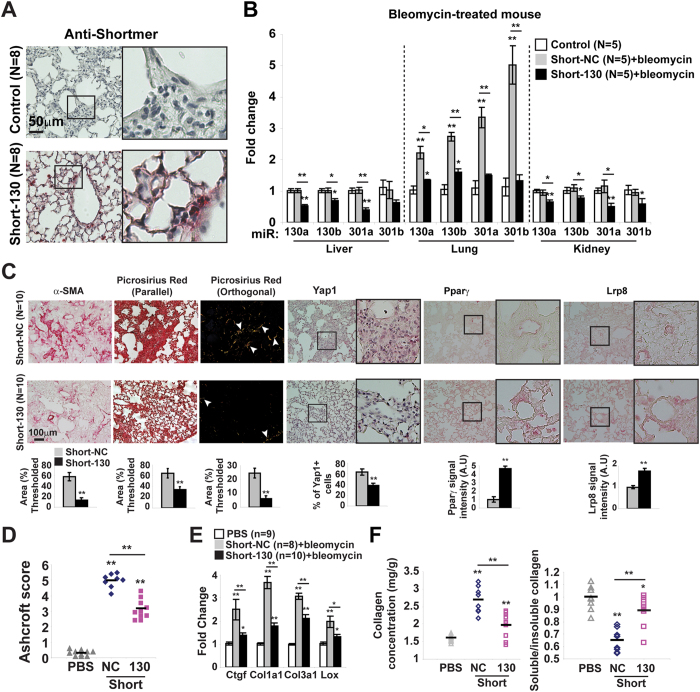
miR-130/301 inhibition ameliorates pulmonary fibrosis. In a model of lung fibrosis (bleomycin-induction), mice were treated with control (Short-NC) or miR-130/301 inhibitor (Short-130) (n = 8/10 per group). Short-130 delivery (n = 8 per group; (**A**) and activity (n = 5 per group; (**B**) were confirmed. Short-130 decreased fibrosis, as assessed by α–SMA and collagen staining (**C**), as well as Ashcroft score (**D**). By *in situ* stain (**C**), Short-130 decreased vascular YAP nuclear localization and increased Pparγ and Lrp8. Short-130 also decreased transcript expression of Ctgf, collagen isoforms, and Lox (**E**) as well as decreased collagen deposition and collagen crosslinking (**F**). Data are expressed as mean ± SEM (*P < 0.05; **P < 0.01).

To determine whether miR-130/301 family members are necessary for control of fibrosis across both pulmonary and liver fibrosis, mice were serially administered control versus Short-130, an antisense oligonucleotide confirmed to inhibit all miR-130/301 family members in cultured cells^12^ and *in vivo* in mouse liver (Fig. 5A,B) and mouse lung (Fig. 6A,B). In both models of pulmonary (bleomycin exposure) and liver (CCl_4_ exposure) fibrosis, Short-130 inhibited miR-130/301 and reversed the down-regulation of miR-130/301 target genes Pparγ and Lrp8 (Figs 5 and 6C), decreased Lox expression, reduced collagen expression and collagen crosslinking (Figs 5C,E,F and 6C,E,F), and reduced Yap nuclear localization (Figs 5C and 6C) and YAP activation, as reflected by CTGF expression (Fig.5E and 6E). In doing so, miR-130/301 inhibition significantly reduced end-stage fibrosis, as assessed by Metavir (Fig. 5D) score and Ashcroft score (Fig. 6D).

### Pharmacologic activation of APOE with LXR agonist GW3965 decreases peri-arteriolar fibrosis and improves lung fibrosis *in vivo*

**Figure 7:**
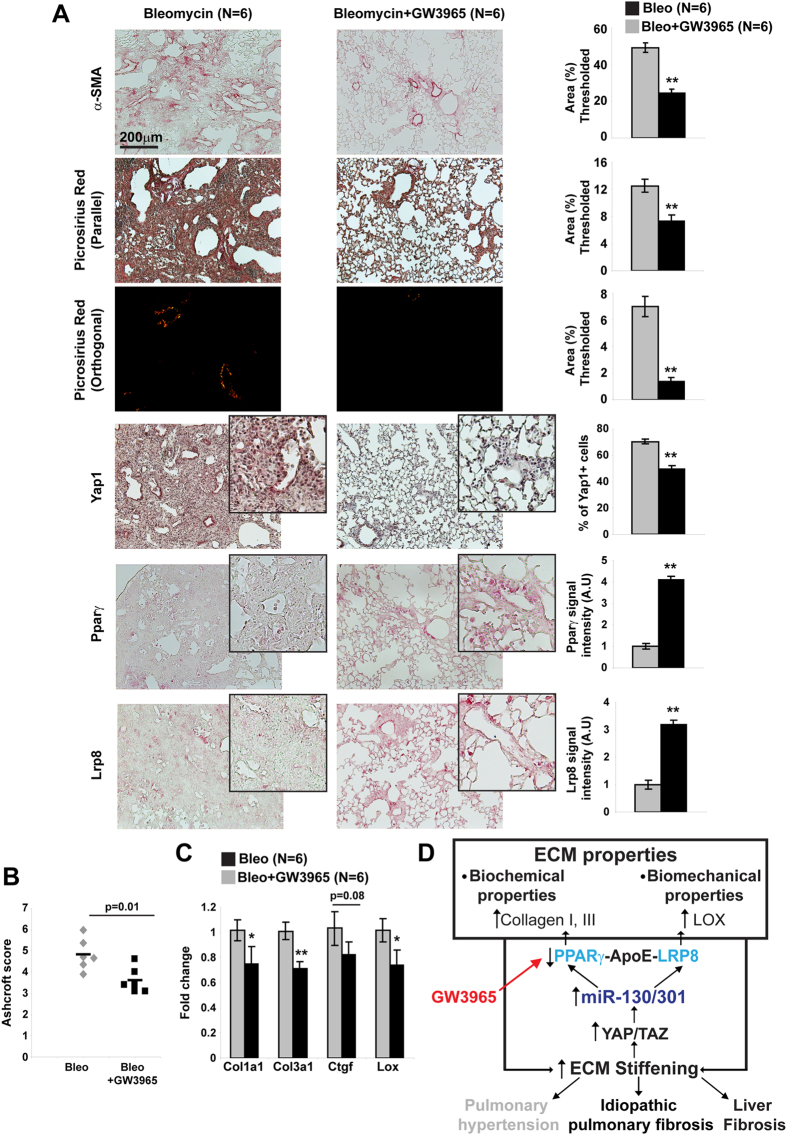
Pharmacological activation of APOE with LXR agonist GW3965 ameliorates pulmonary fibrosis. In a model of lung fibrosis (bleomycin exposure), mice were treated with the LXR agonist GW3965 or control (n = 6 per group) via dietary intake (100 mg/kg). GW3965 ameliorated the degree of lung fibrosis as quantified by α-SMA labeling (**A**) and Aschcroft score (**B**). Picrosirius Red stain and *in situ* staining of mouse lung (**A**) demonstrated that GW3965 blunted bleomycin-mediated increases of collagen crosslinking and YAP, as well as decreased of Lrp8 and Ppar γ expression. **C)** By RT-qPCR, Ctgf, collagen isoforms, and Lox were increased in diseased lung (bleomycin), but GW3965 reduced such increased expression. (**D**) Unifying model of the central role of the YAP/TAZ-miR-130/301 circuit in control of ECM plasticity across related diseases. Data are expressed as mean ± SEM (*P < 0.05; **P < 0.01).

To determine whether downstream ApoE is critical for miR-130/301-induced fibrosis, we attempted to prevent lung fibrosis in bleomycin-exposed mice via treatment with a pharmacological activator of ApoE^13^, the liver-X nuclear hormone receptor agonist GW3965 (Fig. 7). When GW3965 was administered serially after bleomycin exposure, collagen crosslinking (Fig. 7A) was inhibited, leading to decreased indices of lung fibrosis, as reflected by Ashcroft score (Fig. 7B). Consistent with our prior findings of a positive feedback loop in PH involving YAP/TAZ-miR-130/301 that both initiates and results from ECM stiffening^9^, GW3965 treatment also reduced YAP nuclear localization (Fig. 7A) and YAP activation, as reflected by decreased Ctgf expression (Fig. 7C), as well as reversed Pparγ and Lrp8 down-regulation (Fig. 7A). Consequently, we can conclude that the YAP-TAZ-miR-130/301 circuit acts as a master regulator of both fibrotic gene programming and ECM remodeling across multiple pathobiological contexts *in vivo*. Moreover, by offering proof of our *in silico* predictions, these findings identify the fibrotic actions of the miR-130/301 family as a unifying molecular basis for the convergent relationship of seemingly disparate diseases (Fig. 7D).

## Discussion

By using network-based computational modeling and *in vivo* experimentation, we have defined the YAP/TAZ-miR-130/301 molecular circuit and its downstream control of ECM remodeling as a shared and unifying *in vivo* origin of a network of human diseases and physiologic conditions (Fig. 7D). Such identification of “network-based” regulators carries broad implications on the fundamental significance of ECM plasticity in the shared fibrotic origins linking seemingly disparate phenotypes and pathophenotypes. It not only suggests the utility of related pharmacologic strategies (*i.e.*, inhibitors of miR-130/301) for such associated diseases but also the importance of selective use to avoid unintended detrimental consequences of manipulating a pathway so broadly shared among fibrotic processes. Finally, the success of this approach provides much-anticipated experimental evidence for the clinical utility of re-conceptualizing diseases based on systems of shared gene networks and their molecular regulators^14^.

Our results showcase the power of advanced analysis of gene network architecture not only to predict a relevant fibrotic gene “program” shared among related human diseases and physiologic states but also to identify its overarching regulators, such as the miR-130/301 family, across those conditions (Figs 1 and 7D). Currently, experimental evidence and expertise are only just emerging in support of using network theory to guide analysis across human diseases. There is increasing appreciation that “intermediate” phenotypes such as tissue fibrosis are common among human diseases previously thought to develop independently. The molecular overlap of complex human disease states has begun to be interrogated at a systems-wide level^15^,^16^ but our ability to discern the existence of overarching “network-based” regulators across pathologic conditions has remained limited. In that vein, our network analysis also uncovered shared miR-130/301-specific commonalities among diseases that have rarely, if ever, been clinically associated with fibrosis (*i.e.*, Ebola infection, schizophrenia, among others; Table S1). The putative connection of miR-130/301 and ECM biology to schizophrenia is particularly intriguing, as it is a disorder where ECM remodeling in perineuronal nets is already suspected to control final psychiatric manifestations^17^. Furthermore, it is possible that an even more complex and wide-reaching interactome exists among miR-130/301 and additional miRNAs predicted to recognize large portions of the same fibrotic disease network (Fig. 1, Table S2). More detailed analyses of the architecture of that interactome could further reveal as-of-yet undescribed mechanistic connections between this miRNA family, ECM biology, and seemingly unrelated biologic processes. Future experimental validation *in vivo* of these principles would advance the concept of re-classifying diseases based on increasingly available data of molecular signatures that drive the origin of disease^14^.

The coupling of such network-based modeling with experimental validation also facilitated the identification of the YAP/TAZ-miR-130/301 circuit as a broad mediator of mechanotransduction across a variety of tissue beds and disease contexts. Isolated factors such as PPARγ^18^,^19^,^20^ and ApoE^21^,^22^,^23^ as well as their downstream effectors CTGF^24^ and the lysyl oxidase family^25^,^26^,^27^ previously have been implicated in diverse forms of fibrosis. However, their molecular interconnections with upstream regulators have been difficult to define. Separately, a growing appreciation has emerged regarding the activity of YAP/TAZ and microRNAs in general in regulating ECM biology. For instance, YAP/TAZ can drive fibrogenesis and tumorigenesis in an array of transformed tissue^28^,^29^,^30^ such as the liver^31^ and non-transformed tissue including the lung^8^. Additionally, a cohort of so-called “fibromiRs” have been defined in specific cancer-related and non-cancer-related fibrotic diseases, including miR-155^32^, let-7^33^, miR-29^34^,^35^,^36^, miR-21^37^,^38^,^39^, and miR-199^40^,^41^, among others. Yet, only unique miRNAs, such as miR-29^42^ and miR-18^43^ have been found sensitive to YAP activity and tissue mechanics^43^,^44^ in specific contexts such as breast cancer. Unlike prior studies of miR-130/301 family members focusing on specialized conditions such as liver cancer^45^,^46^, breast cancer^47^,^48^, scleroderma^49^, our findings here emphasize the global dysregulation of the miR-130/301 family and its elusive interconnections with the molecular fibrotic machinery of the fibroblast. Given its adjustable, feedback-driven property, the miR-130/301-YAP/TAZ circuit may be responsible, in part, for individualized “tuning” of ECM remodeling observed among different fibrotic disorders. Given our growing appreciation of YAP/TAZ activity resulting from physical stimuli such as shear stress^50^, it is also possible that miR-130/301 and other YAP/TAZ-associated miRNAs may constitute a newly defined set of factors responding to a wide range of physical alterations *(i.e.*, vascular hemodynamics) of the microenvironment in addition to stiffness alone. Identification of these interconnections sheds a new light on the complexity of fibrotic diseases and highlights the attractive candidacy of YAP/TAZ^51^ and miR-130/301^12^,^52^,^53^ for therapeutic targeting.

In regard to the relationship of this disease network to PH specifically, identification of the centrality of the YAP/TAZ-miR-130/301 circuit also provides molecular insight into the heterogenous clinical associations of secondary diseases found with this enigmatic vascular condition^54^. For instance, World Health Organization (WHO) Group I pulmonary arterial hypertension (PAH) is a severe form of PH where disparate secondary diseases are catalogued together based on histopathological parallels rather than molecular similarities (including liver disease and portopulmonary hypertension among others). Alternatively, WHO Group III PH encapsulates a variety of pulmonary diseases including idiopathic pulmonary fibrosis (IPF) where hypoxia has been described as an environmental trigger linking lung pathology to PH. Yet, for a PH-related disease such as idiopathic pulmonary fibrosis (IPF), other unifying yet still unidentified molecular factors and/or cellular pathophenotypes beyond hypoxia likely figure prominently. Our data implicating the remodeling of the ECM among this network of diseases form the foundation of two non-mutually exclusive models of how diseases such as pulmonary fibrosis and fibrotic liver diseases may intersect with PH. First, these diseases may develop in a cell autonomous fashion, driven by separate injuries [such as hypoxia, inflammation, and specific genetic mutations linked to PH^12^] that induce miR-130/301 in distinct tissue beds. Thus, it is tempting to speculate that genetic mutations linked to PH and miR-130/301^9^ may also contribute to separate fibrotic lung and liver diseases. A second model implicates non-cell autonomous methods of activating this molecular feedback loop among distinct anatomic tissue beds. For instance, in WHO Group III PH associated with pulmonary fibrosis, our results indicate that, independent of hypoxia, parenchymal fibrosis may activate the YAP/TAZ-miR-130/301 circuit in adventitial fibroblasts and perhaps other related mesenchymal stem cells^55^, thus accelerating vascular stiffness. Moreover, given increased miR-130/301 levels in plasma of PH patients^12^,^56^, pathogenic transfer and endocrine signaling of miR-130/301 between lung and liver is an intriguing possibility. Both models may be active, and the identification of central factors common to all diseases provides a much needed molecular landmark to decipher additional complexities of disease interconnection.

The identification of a fibrotic miRNA-dependent gene program shared across multiple diseases carries broad clinical ramifications beyond disease re-classification. At the diagnostic level, molecular screening for this fibrotic signature could facilitate the ability to identify persons at risk for such a related “syndrome” of diseases. Alternatively, if certain fibrotic disease states exist (*i.e.*, potentially at earlier time points of disease progression or specific clinical subtypes) where individual molecules are activated in isolation such as miR-130/301 or ApoE, more tailored therapy could be attempted and may be effective in those cases. As such, pre-treatment biopsies could be helpful, although assessment would require simultaneous RNA and protein assays – protocols that are not yet widespread in clinical practice. Despite these challenges, a potential avenue for personalized therapy and diagnosis could be envisioned.

At the therapeutic level, the efficacy of targeting individual fibrotic enzymes, such as matrix metalloproteinases (MMPs), has been suboptimal, especially when tested at the clinical stage^57^,^58^. Especially since YAP/TAZ and miR-130/301 appear to activate a positive feedback loop for robust ECM stiffening at least in PH^9^, a pharmacologic combination of miR-130/301 inhibition via shortmers^59^ in addition to manipulating downstream miR-130/301-dependent pathways such as LXR/APOE activity (Fig. 7) offers a rational avenue for cooperative therapeutic targeting of fibrosis. Yet, because of the systems-level actions of miR-130/301 on multiple vascular cell phenotypes^9^,^12^,^52^, proper clinical use of miR-130/301 inhibitors may have caveats. First, because fibrosis and matrix production also have positive and adaptive attributes in certain conditions (*i.e.*, normal wound healing and organ development), inhibition of miR-130/301 may have substantial pitfalls, if used in non-selective contexts. Second, such pitfalls may be exaggerated by the widespread but cell-type specific functions of this miRNA family beyond the fibroblast, presumably dictated by a non-identical cohort of miR-130/301-specific target genes active in differing cell types^9^. Nonetheless, selective pharmacologic targeting of the fibroblast in specific disease conditions holds promise, and future biological confirmation of the broader, network-based actions of miR-130/301 family could further guide such a systems pharmacology approach to targeting fibrosis which has not yet been pursued in great depth.

Together, the results herein define the control of a fibrotic gene program by the YAP/TAZ-miR-130/301 circuit, shared among a network of related diseases. These findings re-define our conception of the elusive molecular origins of these diseases, thus offering much-needed molecular diagnostic and therapeutic opportunities. Moreover, by combining advanced network analyses with experimental interrogation, our results provide a roadmap to study systems-level cooperativity pervasive among miRNAs and the human disease network, in general. Such future work has the potential to uncover additional hidden yet fundamental links present in seemingly unrelated pathology.

## Methods

### Collection and Analysis of Expression Array Data

In order to include transcriptomic data in our statistical assessment of miRNA activity in fibrotic states, we compiled a set of 137 expression arrays analyzing human tissue in a variety of disease conditions and physiologic conditions (see also Table S1). Publically available arrays were found in the Gene Expression Omnibus (GEO) database, filtered to include only analyses performed on untreated, affected human tissue and drawn blood (as compared with matched, non-diseased control samples of the same tissue type), with one array per tissue type per disease or physiologic state. Where available, we selected studies in which healthy tissue from living, age-matched patients was available, though for the sake of sample size, we also made use of studies in which postmortem biopsies were used, if no other data was available. Conditions were excluded for which the only available tissue samples were not directly relevant to the physiology in question, as in the case of pulmonary hypertension (PH), where only peripheral blood samples from PH patients had been analyzed, as opposed to more disease-relevant solid tissue from the lung. The final dataset represented 137 expression array sets representing 105 distinct conditions, and included a broad cross-section of human pathology. We selected those genes in each array set that showed a fold-change > ± 2.0 and a p-value < 0.05, and cross-referenced the resulting gene set with the consolidated interactome to form a unique gene network.

Only the top 250 genes (by fold-change) were selected in cases where the number of significantly differentially expressed genes exceeded this cutoff. These networks were then expanded to include well-connected first-degree interactors, as described above. Such truncation was necessary to optimize our ability to discern biologically meaningful molecular overlap, due to the inherent noise and effect saturation in comparing excessively large gene sets. First, because expression array analyses are susceptible to noise, we decreased the number of false-positives present in our network by including only those genes that show the greatest amount of modulation in a given condition. Second, because metrics such as the spanning score are based largely on the overlap of miRNA target pools with the network in question, an excessively large gene network would present a situation in which the upper end of the score spectrum can become saturated, thus increasing the difficulty in differentiating the relative influence of top-scoring miRNAs from one another. While some information can be lost in such truncation (leading to higher false-negative rate), we postulated that focusing on genes with the largest expression changes would provide a reasonable representation of the key, systems-level alterations in each diseased tissue. Furthermore, the process of first thresholding and then expanding the network by adding in well-connected first-degree interactors provided an additional method (along with the standard fold-change and p-value cutoffs described above) for isolating and amplifying the molecular signal of these putative systems-level changes. Thus, overall, we generated a conservative representation of the available expression data, favoring more accurate networks with few false-positives over networks that are fully comprehensive.

For each of the 137 networks, we determined (a) the fraction of the network that overlapped with the fibrosis signature, and (b) the ranked list of miRNAs (by spanning score) that influenced the network. In order to isolate miRNAs that are specifically relevant to fibrosis (and not simply influential across all networks by virtue of having a large and well-connected target pool), we grouped networks into four cohorts according to the size of their overlap with the fibrosis signature. We then ranked miRNAs based on a one-way ANOVA means comparison test for their assigned spanning score rank for the networks in each cohort. miRNAs that scored well by this metric had higher average spanning scores in strongly fibrotic disease groups, relative to their performance overall. Because miRNA-target predictions were generated based on seed sequence matching^60^, our spanning scores group individual miRNAs into families with identical seed regions.

Upon identifying the miR-130/301 family as a critical regulator of fibrosis across contexts, we ranked each of the 137 networks according to (a) its overlap with the fibrosis network and (b) the spanning score rank assigned to miR-130/301 in that context. This was quantified as the average of two values: (1) the fraction of network genes that were shared with the fibrosis network and (2) the fraction of the highest possible rank achieved by miR-130/301 [1 – rank_130_/rank_MAX_]. This ranking identified networks that contained significant fibrotic components and were heavily influenced by this miRNA family.

### *In situ* hybridization

The protocol for *in situ* hybridization for miRNA detection was based on a prior report^12^. Specifically, 5 μm tissues sections were probed using a 3′ fluorescein isothiocyanate (FITC) labeled miRCURY LNA hsa-miR-130a detection probe (Exiqon; 5′-ATGCCCTTTTAACATTGCACTG-3′). The miRCURY LNA scramble-miR probe was used as negative control. Following re-hydration (Sigma) tissues were formaldehyde-fixed (4% formaldehyde, Sigma) before inactivation of endogenous enzymes by acetylation buffer [873 uL of triethanolamine (Sigma) and 375 uL acetic anhydride (Fisher) in 75 ml distilled water]. Probe annealing (25 nM LNA probe) was performed in hybridization buffer (Sigma, H7782) for 16 hours at RNA-Tm-22 °C (62 ^o^C). Following serial washes with 2X SSC, 1X SSC, and 0.5X SSC (Sigma) at 62 ^o^C, immunolabeling was performed with an anti-FITC biotinconjugated antibody for overnight at 4 ^o^C (1:400; Sigma-Aldrich). For detection, development was achieved by adding streptavidin-biotinylated alkaline phosphatase complex (Vector Labs) followed by Nitro blue tetrazolium chloride/5-Bromo–4-chloro–3–indolyl phosphate substrate solution (NBT/BCIP, Roche), and positive staining was evident by a blue color. MiR-130a expression was quantified in the vascular wall of 15–20 pulmonary arteries or for liver tissue in 10 random 20x fields per animal using ImageJ software (NIH).

For co-detection of proteins and miRNA, after probe hybridization and following serial washes with SCC immunolabeling was performed with anti-α–SMA (1/500; Sigma-Aldrich), anti-vimentin (1/250; Abcam) and/or anti-Desmin (1/1000; Abcam) antibody for overnight at 4 ^o^C. After 3 washes in TBS Tween 0.1% slides were incubated with donkey anti-mouse and donkey anti-rabbit Alexa-conjugated antibody (Alexa 568 and Alexa 647) for 1 hour at room temperature. After 3 washes in TBS/Tween 0.1%, slides were mounted with anti-fading medium with DAPI (Vectashields, Vector).

## Animals 

### All animal treatments and analyses were conducted in a controlled and non-blinded manner.

#### Lung fibrosis model

Age-matched male C57BL/6 mice (7–8 weeks old, substrain N) were exposed to 0.035 U of bleomycin via oropharyngeal route. In the control group, saline (PBS) was administered via the same route. Mice were euthanized 14 days or 21 days after bleomycin administration.

#### Liver fibrosis model

For chronic CCl_4_–induced liver fibrosis, age matched male C57BL/6 mice (7–8 weeks old, substrain N) were injected (intra-peritoneal) with 1 mL per kg body weight sterile CCl_4_ in a 1:5 ratio in corn oil or corn oil alone (control) every 5 days for 4–6 weeks. Livers were harvested 72 h after the last injection.

#### Inhibition of miR-130/301 in a mouse model of pulmonary fibrosis

Eight-week-old mice (C57Bl6) were injected with bleomycin (1.5U/kg Sigma Aldrich) followed by 10 intraperitoneal injections (every 2 days) of control or miR-130/301 shortmer oligonucleotides, designed as antisense inhibitors recognizing the seed sequence of this miRNA family (20 mg/kg/dose; Regulus). Shortmer generation was previously described^12^. Two days after the last injection, lung tissue was harvested for RNA extraction or paraffin embedding.

#### Forced expression of miR-130a in liver tissue of mice

Eight-week-old mice (C57BL/6) were injected by intraperitoneal route every 3 days during 6 weeks with 1 nmol of miR-control (pre-miR-NC) or miR-130a (pre-miR-130a) (Thermo Fisher Scientific/Life Technologies) mixed in 100 μl PBS solution containing 5% Lipofectamine 2000 (Thermo Fisher Scientific/Life Technologies). These injections were accompanied by 6 intraperitoneal injections (once every seven days) of suboptimal dose of CCl4 (0.1mg/kg body weight) or vehicle (Corn oil, Sigma-Aldrich). Two days after the last injection, liver, lung and kidney tissues were harvested for RNA extraction or paraffin embedding.

#### Inhibition of miR-130/301 in a mouse model of liver fibrosis

Eight-week-old mice (C57Bl6) were injected with CCl_4_ (1mL per kg of body weight) every 5 days accompanied by intraperitoneal injections (every 2 days) of control or miR-130/301 shortmer oligonucleotides (20mg/kg/dose; Regulus). Two days after the last injection, liver tissue was harvested for RNA extraction or paraffin embedding.

#### Treatment of mice with oral ingestion of the liver-X nuclear hormone receptor (LXR) agonist GW3965

To determine the effects of the LXR agonist GW3965 (Sigma-Aldrich) and consequent APOE induction on lung fibrosis, as previously described^13^, mice were exposed to bleomycin (as described above) and simultaneously assigned to control chow or chow supplemented with GW3965 (Research Diets, *Inc*.) at doses of 100 mg of drug per kilogram of mouse per day (based on average daily intake of 3.5 gm of chow). After two weeks, harvest of lung tissue was performed for RNA/protein extraction or paraffin embedding.

#### Study approval and ethics statement

All human and animal experiments were carried out in accordance with the approved guidelines listed below. Specifically, all animal experiments were approved by the Harvard Center for Comparative Medicine. All experimental procedures involving the use of human tissue were approved by Institutional Review Boards at Partners Healthcare, Boston Children’s Hospital, National Institutes of Health, as well as the New England Organ Bank. Ethical approval for this study conformed to the standards of the Declaration of Helsinki. For formalin-fixed paraffin embedded lung samples and flash frozen samples, human idiopathic pulmonary fibrosis (IPF) specimens were collected from discarded surgical samples following lung transplantation. Control lung samples were collected in cases where non-IPF donor lungs were declined for transplantation. All subjects provided informed consent prior to tissue procurement. No additional clinical data were available. As previously described^61^, explant lung tissue samples were procured within one hour of the transplant procedure, tissue slides were obtained from paraffin-embedded blocks of explants fixed in 4% paraformaldehyde, snap frozen or stored in RNA preservative. For formalin-fixed paraffin embedded liver samples, informed consent was obtained for standard-of-care wedge liver biopsy samples, collected from patients (Massachusetts General Hospital) undergoing weight loss surgery for the purpose of evaluating for nonalcoholic steatohepatitis (NASH). For this study, specimens were analyzed from unused surgical specimens that had previously been evaluated by a pathologist blinded to clinical data and scored as previously described^62^,^63^.

## Statistics. 

The number of animals in each group was calculated to measure at least a 20% difference between the means of experimental and control groups with a power of 80% and standard deviation of 10%. The number of unique patient samples for this study was determined primarily by clinical availability. *In situ* expression/histologic analyses of both rodent and human tissue, and pulmonary vascular hemodynamics in mice and rats were performed in a blinded fashion. Numerical quantifications for physiologic experiments using rodents or human reagents represent mean ± standard error of the mean (SEM). Paired samples were compared by a 2-tailed student’s *t* test. A P-value less than 0.05 was considered significant. Correlation analyses were performed by Pearson correlation coefficient calculation.

## Additional Information

**How to cite this article**: Bertero, T. *et al.* A YAP/TAZ-miR-130/301 molecular circuit exerts systems-level control of fibrosis in a network of human diseases and physiologic conditions. *Sci. Rep.* **5**, 18277; doi: 10.1038/srep18277 (2015).

## Supplementary Material

Supplementary Information
