# Supplementary material for: A YAP/TAZ-miR-130/301 molecular circuit exerts systems-level control of fibrosis in a network of human diseases and physiologic conditions
Source: Sci Rep. 2015 Dec 15;5:18277. doi: 10.1038/srep18277 (PMC4678880; doi:10.1038/srep18277)
Supplement: Supplementary Information [file srep18277-s1.pdf]

Supplemental

A YAP/TAZ-miR-130/301 molecular circuit exerts systems-level control of fibrosis in a network of human diseases and physiologic conditions

Thomas Bertero, Katherine A. Cottrill, Sofia Annis, Balkrishen Bhat, Bernadette R. Gochuico, Juan C. Osorio, Ivan Rosas, Kathleen J. Haley, Kathleen E. Corey, Raymond T. Chung, B. Nelson Chau, and Stephen Y. Chan

Supplemental Methods

Network Construction

The initial fibrosis network was constructed as previously described (Bertero *et al.*, submitted manuscript). Briefly, we manually curated a set of 133 genes known fibrotic genes focusing only on genes known to play a causative role in tissue fibrosis. Interactions between curated genes were annotated according to a master list of protein-protein, protein-DNA, kinase-substrate, and metabolic interactions, drawn from several consolidated databases, referred to here as the "consolidated interactome" [1]. In order to capture any additional fibrotic genes that may have been missed in our initial curation, we also incorporated a select number of non-curated genes ("fibrosis interactors") demonstrated to interact with a significant number of genes in our curated set. The resulting fibrosis network contained 350 nodes and 1459 edges, with a largest connected component of 339 nodes.

Network Clustering

Clustering was performed using the Louvain method for community detection [2], as implemented in the NetworkX package for Python 3.3. The final partition was selected so as to maximize modularity in the graph.

miRNA Target Prediction

miRNA target prediction was performed using the TargetScan 6.2 (Conserved) algorithm [3]. The TargetScan algorithm detects mRNA with conserved complementarity to the

“seed” (nucleotides 2-7) of a given miRNA. Because of this, miRNA that share a seed are grouped together as a family and regarded as a single unit by the algorithm. For this reason, we do not distinguish between miRNA belonging to the same family in any of our statistical analyses.

miRNA Spanning Score

In order to rank the influence of miRNA families on the fibrosis network (and other disease networks), we ranked miRNAs based on their “spanning score”. As previously described [4], this metric scores a miRNA family on three criteria: (1) the number of network genes which it targets, (2) the number of network clusters in which its targets reside, and (3) the hypergeometric p-value for the overlap of its target pool with the network. Each of these criteria is scored relatively, as a fraction of the maximum value achieved by any miRNA in our dataset for the network under consideration. This method provides a holistic assessment of the influence of a miRNA family on a given network of genes, considering not only the size, but also the spread and statistical significance of its target pool within the network.

Messenger RNA and miRNA extraction

Tissues were homogenized in 1 ml of QiaZol reagent (Qiagen). Total RNA content, including small RNAs, was extracted using the miRNeasy kit (Qiagen) according to the manufacturer’s instructions. Total RNA concentration was determined using a ND-1000 micro-spectrophotometer (NanoDrop Technologies).

Quantitative RT-PCR of mature miRNAs

Mature miRNA expression was evaluated using TaqMan MicroRNA Assays (Thermo Fisher Scientific) and the Applied Biosystems 7900HT Fast Real Time PCR device. Expression levels were normalized to RNU48 or snoR55 for human or mouse experiments, respectively, and calculated using the comparative Ct method ($2^{-\Delta\Delta Ct}$). Primers (Thermo Fisher Scientific) included: miR-130a (Cat.# 000454); miR-130b (Cat.# 000456); miR-301a (Cat.# 000528); miR-301b (Cat.# 002392); miR-454 (Cat.# 002323); RNU48 (Cat.# 001006); and snoR55 (Cat.# 001228).

Quantitative RT-PCR of messenger RNAs

Messenger RNAs were reverse transcribed using the Multiscript RT kit (Life Technologies) to generate cDNA. cDNA was amplified via fluorescently labeled Taqman primer sets using an Applied Biosystems 7900HT Fast Real Time PCR device. Fold-change of RNA species was calculated using the formula ($2^{-\Delta\Delta Ct}$). Based on our empiric experience of stability of actin across a multitude of physiologic and pathophysiologic conditions and across multiple tissue type, calculations were normalized to actin expression which did not vary by more than 0.5 cycle lengths with any comparison. Mouse primers (Thermo Fisher Scientific) included: Actin (Cat.# Mm00607939_s1**);** Col1a1 (Cat.# Mm00801666_g1); Col3a1 (Cat.# Mm01254476_m1); Lox (Cat.# Mm00495386_m1); Ctgf (Cat.# Mm01192932_g1). Human primers (Thermo Fisher Scientific) included: Actin (Cat.# Hs99999903_m1); COL1A1 (Cat.# Hs00164004_m1); COL3A1 (Cat.# Hs00943809_m1); LOX (Cat.# Hs00942480_m1); CTGF (Cat.# Hs01026927_g1).

Lung and liver tissue harvest

In mice, after physiological measurements by direct right ventricular puncture, the pulmonary vessels were gently flushed with 1 cc of saline to remove the majority of blood cells, prior to harvesting cardiopulmonary tissue. The heart was removed, followed by dissection and weighing of the right ventricle (RV) and of the left ventricle + septum (LV+S). Organs were then harvested for histological preparation or flash frozen in liquid N2 for subsequent homogenization and extraction of RNA and/or protein. To further process lung tissue specifically, prior to excision, lungs were flushed with PBS at constant low pressure (~10mmHg) via right ventricular cannulation, followed by tracheal inflation of the left lung with 10% neutral-buffered formalin (Sigma-Aldrich) at a pressure of ~20cm H_2O. After excision and 16 hours of fixation in 10% neutral-buffered formalin at 25°C, lung and liver tissues were paraffin-embedded via an ethanol-xylene dehydration series, before being sliced into 5µm sections (Hypercenter XP System and Embedding Center, Shandon).

Immunohistochemistry of lung and liver

Lung and liver sections (5μm) were deparaffinized and high temperature antigen retrieval was performed, followed by blocking in TBS/BSA 5%, 10% goat serum and exposure to primary antibody and biotinylated secondary antibody (Vectastain ABC kit, Vector Labs). A primary antibody against YAP1 (#4912; 1/200) was obtained from Cell Signaling. Primary antibodies against, LRP8 (ab115196; 1/100), α-SMA (1/400) were purchased from Abcam. Primary antibodies against PPARγ (sc-7273; 1/50) was

purchased from Santa Cruz. Primary antibody against anti-shortmer (E5746-B3A; 1/1000) was provided by Regulus Therapeutics, as previously described [4]. In most cases, color development was achieved by adding streptavidin-biotinylated alkaline phosphatase complex (Vector Labs) followed by Vector Red alkaline phosphatase substrate solution (Vector Labs). Levamisole was added to block endogenous alkaline phosphatase activity (Vector Labs). Pictures were obtained using an Olympus Bx51 microscope. For liver tissue and pulmonary tissue, 10 random 20x fields per animal were analyzed. Intensity of staining was quantified using ImageJ software (NIH). Degree of pulmonary fibrosis was assessed in paraffin-embedded lung sections stained for α–SMA by Ashcroft score [5]. Degree of liver fibrosis was assessed in paraffin-embedded liver sections stained for α–SMA by Metavir score [5]. All measurements were performed blinded to condition.

Picrosirius Red stain and quantification

Picrosirius Red stain was achieved through the use of 5μm paraffin sections stained with 0.1% Picrosirius Red (Direct Red80, Sigma) and counterstained with Weigert's hematoxylin to reveal fibrillar collagen. The sections were then serially imaged using with an analyzer and polarizer oriented parallel and orthogonal to each other. Microscope conditions (lamp brightness, condenser opening, objective, zoom, exposure time, and gain parameters) were constant throughout the imaging of all samples. A minimal threshold was set on appropriate control sections for each experiment in which only the light passing through the orthogonally-oriented polarizers representing fibrous structures (*i.e.*, excluding residual light from the black background) was included. The threshold

was maintained for all images across all conditions within each experiment. The area of the transferred regions that was covered by the thresholded light was calculated and at least 10 sections per condition were averaged together (Image J software).

Measurement of collagen content in lung and liver tissue

This protocol was adapted from a previously published protocol [6]. Mouse lung or liver tissue was weighed, minced, and incubated in 0.5 M acetic acid at 4°C. After overnight digestion, the acetic acid-soluble and insoluble fractions were isolated by centrifugation. The soluble fraction was stored at -80°C, while the insoluble fraction was digested by overnight incubation in 6M hydrochloric acid at 85°C. Concentrations of soluble and insoluble (gelatinous) collagen fractions were determined using a Sircol Soluble Collagen Assay Kit (Biocolor) with a colorimetric reaction (measured at 550 nm) and a provided collagen reference standard curve.

Supplemental Tables

TABLE S1: Networks ranked by their interconnectedness with the fibrosis network and the miR-130/301 family. Distinct networks of various human conditions (n=137) were constructed based on expression profiling of affected human tissue. These data were generated from the Gene Expression Omnibus (GEO) database. Diseases were ranked based on the influence of the miR-130/301 family (by spanning score rank) and their percentage overlap with the fibrosis network (quantified as the average of two values: (1) the fraction of network genes that are fibrotic and (2) the fraction of the highest possible rank achieved by miR-130/301 [1 – $rank_{130}/rank_{MAX}$]).

Index	Condition	Tissue	Percentage Fibrotic	Network Size	miR-130/301 Rank	GEO ID
1	Helicobacter Pylori Infection	Corpus Gastric Biopsy	0.677	130	10	GDS625
2	Cerebral Palsy	Gracilis Muscle	0.414	500	4	GDS4353
3	Zaire Ebolavirus Infection	Macrophage	0.413	326	5	GDS4356
4	Alcoholic Hepatitis	Liver Tissue	0.485	132	17	GDS4389
5	Pterygium	Conjunctiva	0.44	386	14	GDS1758
6	Pituitary Gonadotrope	Tumor Tissue	0.388	500	9	GDS4275
7	Pituitary Null Cell Tumor	Tumor Tissue	0.336	500	3	GDS4275
8	Helicobacter Pylori Infection	Antumn Gastric Biopsy	0.388	258	11	GDS625
9	Idiopathic Thrombocytope nic Purpura, Remission	CD3 Cells	0.328	500	3	GDS390
10	Tuberous Sclerosis Complex Periungual Fibroma	Fibroblasts	0.36	500	8	GDS3281

Index	Condition	Tissue	Percentage Fibrotic	Network Size	miR-130/301 Rank	GEO ID
11	Idiopathic Pulmonary Fibrosis	Whole Lung	0.335	478	10	GDS1252
12	Acute Rotavirus Infection	Peripheral Blood Mononuclear Cells	0.281	420	2	GDS2048
13	Familial Combined Hyperlidemia	Peripheral Blood	0.328	378	12	GDS946
14	Autism Fragile X Type	Lymphoblastoid Cells	0.26	500	3	GDS2824
15	Schizophrenia	Postmortem Prefrontal Cortex	0.357	140	18	GDS3345
16	Tuberous Sclerosis Complex Angiofibroma	Fibroblasts	0.3	500	10	GDS3281
17	Dermatomyositis	Skeletal Muscle Biopsy	0.328	500	15	GDS2153
18	ER Negative Breast Cancer	Tumor Tissue	0.232	500	1	GDS3716
19	Idiopathic Pulmonary Fibrosis	Fibroblasts	0.311	446	15	GDS1012
20	Bipolar Disorder	Postmortem Prefrontal Cortex	0.422	128	33	GDS3345
21	Head and Neck Squamous Carcinoma	Tumor Biopsy	0.382	404	28	GDS3838
22	ER Positive Breast Cancer	Tumor Tissue	0.252	500	10	GDS3716
23	UV Exposure	Skin Fibroblasts	0.376	500	29	GDS400

Index	Condition	Tissue	Percentage Fibrotic	Network Size	miR-130/301 Rank	GEO ID
24	Morbid Obesity	Omental Adipose Tissue	0.244	500	9	GDS3679
25	Idiopathic Thrombocytopenic Purpura, Active	CD3 Cells	0.256	500	11	GDS390
26	Uterine Fibroid	Uterine Leiomyoma Tissue	0.32	500	21	GDS484
27	Air Pollution Exposure, Child	Peripheral Blood	0.256	500	12	GDS3325
28	Scleroderma-Associated Pulmonary Fibrosis	Fibroblasts	0.267	480	15	GDS1012
29	Protein Deficiency	Skeletal Muscle	0.197	426	5	GDS2868
30	Aging	Frontal Cortex Male	0.26	500	16	GDS707
31	Primary Immunodeficiency Syndrome	B Cells	0.192	500	6	GDS2723
32	Gastric Cancer	Tumor Tissue	0.36	500	32	GDS1210
33	Idiopathic Amyotrophic Lateral Sclerosis	Spinal Cord Gray Matter	0.38	500	36	GDS412
34	Smoking	Macrophage	0.248	500	16	GDS3496
35	Non-Melanoma Skin Cancer	Actinic Keratotic Lesion	0.208	500	10	GDS2200
36	Osteoarthritis	Chondrocytes, Monolayer	0.232	500	14	GDS3758

Index	Condition	Tissue	Percentage Fibrotic	Network Size	miR-130/301 Rank	GEO ID
		Culture				
37	MELAS Syndrome	Muscle Biopsy	0.217	498	12	GDS1065
38	Melanoma In Situ	Biopsy	0.196	500	9	GDS1989
39	Aging	Frontal Cortex Female	0.276	500	22	GDS707
40	Typhus Infection	HMEC1 Endothelial Cells	0.228	500	15	GDS3848
41	Juvenile Polyarticular Rheumatoid Arthritis	Synovial Fluid	0.384	500	39	GDS711
42	Cerebral Palsy	Semitendinos us Muscle	0.328	500	31	GDS4353
43	Papillary Thyroid Cancer	Tumor Tissue	0.236	500	17	GDS1732
44	Severe Acute Respiratory Syndrome	Peripheral Blood Mononuclear Cells	0.316	500	30	GDS1028
45	Rheumatoid Arthritis	Synovial Tissues	0.296	500	27	GDS2126
46	Progressive External Opthalmoplegi a	Muscle Biopsy	0.176	500	9	GDS1065
47	HIV-1 Infection	Macrophage	0.293	198	28	GDS3511
48	Parkinsons Disease	Inferior Olivary Nucleus	0.26	500	23	GDS4154
49	Mild Cystic Fibrosis	Nasal Respiratory Epithelium	0.188	500	12	GDS2143

Index	Condition	Tissue	Percentage Fibrotic	Network Size	miR-130/301 Rank	GEO ID
50	Breast Cancer	Stromal Tissue	0.35	40	37	GDS4114
51	Chlamydia Pneumonia Infection	Dendritic Cells	0.236	500	24	GDS3573
52	Essential Thrombocythe mia	Platelet	0.212	500	22	GDS1376
53	Hepatitis C Virus Infecton	Huh8 Hepatoma Cells	0.346	182	44	GDS4160
54	Diabetic Nephropathy	Glomeruli	0.332	500	42	GDS961
55	Teratozoosper mia	Sperm Cells	0.08	500	5	GDS2697
56	Wilms Tumor	Tumor Tissue	0.264	500	34	GDS1791
57	Juvenile Pauciarticular Rheumatoid Arthritis	Synovial Fluid	0.4	500	55	GDS711
58	Scott Syndrome	B Lymphoblast	0.366	454	50	GDS1320
59	Vulvar Intraepithelial Neoplasia	Lesion Biopsy	0.104	500	10	GDS2418
60	Acute Myeloid Leukemia	Bone Marrow	0.187	182	23	GDS3057
61	Pediatric Obesity	Omental Adipose Tissue	0.24	500	32	GDS3688
62	Exposure To Laminar Shear Stress	Cultured HUVECs	0.2	500	26	GDS3868
63	Juvenile	Skeletal	0.084	500	9	GDS3417

Index	Condition	Tissue	Percentage Fibrotic	Network Size	miR-130/301 Rank	GEO ID
	Dermatomyositis, Long Duration	Muscle				
64	Amyotrophic Lateral Sclerosis	Muscle Biopsy	0.06	500	6	GDS2855
65	Oligodendroglioma	Tumor Tissue	0.02	500	1	GDS1813
66	Non-Melanoma Skin Cancer	Squamous Cell Carcinoma Lesion	0.316	500	47	GDS2200
67	Myelodysplastic Syndrome	Bone Marrow CD34 Progenitor Cells	0.32	500	48	GDS1392
68	Juvenile Dermatomyositis, Short Duration	Skeletal Muscle	0.068	500	10	GDS3417
69	Juvenile Spondylo-arthropathy	Synovial Fluid	0.3	500	46	GDS711
70	Endometrioma	Endometrial Tissue	0.024	500	4	GDS3975
71	Sepsis	Skeletal Muscle	0.212	424	33	GDS3463
72	Presymptomatic Malaria	Peripheral Blood Mononuclear Cells	0.244	500	38	GDS2362
73	Cigarette Smoking, Female	Oral Mucosa	0.08	50	13	GDS3709
74	Acne	Inflammatory Papules	0.26	470	41	GDS2478

Index	Condition	Tissue	Percentage Fibrotic	Network Size	miR-130/301 Rank	GEO ID
75	Non-Union Skeletal Fracture	Callous Bone	0.012	500	4	GDS369
76	Epstein-Barr Positive Nasopharangeal Carcinoma	Tumor Tissue	0.26	500	44	GDS3610
77	Emphysema	Lung Tissue	0.296	500	50	GDS737
78	Respiratory Syncytial Virus Infection	Bronchial Epithelial Cell Culture	0.204	500	36	GDS2023
79	Ulcerative Colitis	Descending Colon, Uninflamed	0.06	500	14	GDS3268
80	Idiopathic Myelofibrosis	Hematopoietic CD34 Stem Cells	0.232	500	41	GDS2397
81	Multiple Sclerosis	Brain Lesion	0.08	500	18	GDS4218
82	Alzheimers	Hippocampal CA1 Gray Matter	0.212	500	39	GDS4136
83	Spastic Paraplegia	Muscle Biopsy	0.012	500	9	GDS2855
84	Autosomal Dominant Monocytopenia	Polymorphonuclear Cells	0.124	500	27	GDS3820
85	Familial Amyotrophic Lateral Sclerosis	Spinal Cord Gray Matter	0.22	500	42	GDS412
86	Type 2 Diabetes	Pancreatic Islets	0.076	500	20	GDS4337
87	Preeclampsia	Placental Tissue	0.136	500	31	GDS3467

Index	Condition	Tissue	Percentage Fibrotic	Network Size	miR-130/301 Rank	GEO ID
88	Morbid Obesity	Subcutaneous Adipose Tissue	0.297	128	57	GDS3679
89	Carious Pulpal Tissue	Dental Pulp	0.264	500	53	GDS1850
90	Atopic Dermatitis	Lesional Skin	0.012	500	15	GDS2382
91	Acute Myeloid Leukemia	Peripheral Blood	0.225	376	48	GDS3057
92	Acute Quadriplegic Myopathy	Muscle Biopsy	0.112	500	32	GDS2855
93	Vertical Growth Phase Melanoma	Lesion Biopsy	0.04	500	22	GDS1989
94	Invasive Ductal Carcinoma	Tumor Biopsy	0.092	500	30	GDS3853
95	Aldosterone-Producing Adenoma	Tumor Biopsy	0.032	500	22	GDS2860
96	Osteoarthritis	Chondrocytes, Matrix Culture	0.056	500	26	GDS3758
97	Becker Muscular Dystrophy	Muscle Biopsy	0.112	500	35	GDS2855
98	Inflammatory Dilated Cardiomyopathy	Endomyocardium	0.012	500	20	GDS2154
99	Obesity, Female	Cultured Abdominal Adipocytes	0.232	392	54	GDS1497
100	Clear Cell Renal Cell Carcinoma Stage II	Tumor Tissue	0.012	500	21	GDS2881

Index	Condition	Tissue	Percentage Fibrotic	Network Size	miR-130/301 Rank	GEO ID
101	Severe Cystic Fibrosis	Nasal Respiratory Epithelium	0.008	500	21	GDS2143
102	Clear Cell Renal Cell Carcinoma Stage I	Tumor Tissue	0.02	500	23	GDS2881
103	Malignant Pleural Mesothelioma	Pleura	0.052	500	29	GDS1220
104	Polycystic Ovary Syndrome	Granulosa Cells	0.14	500	44	GDS4399
105	Abdominal Aortic Aneurysm	Abdominal Aorta	0.172	500	49	GDS2838
106	Testicular Seminoma	Tumor Biopsy	0.144	500	45	GDS2842
107	Depression	Postmortem Prefrontal Cortex	0.113	106	41	GDS3345
108	Parkinson’s Disease	Dorsal Motor Nucleus of the Vagus	0.176	500	51	GDS4154
109	Symptomatic Malaria	Peripheral Blood Mononuclear Cells	0.18	500	52	GDS2362
110	Influenza Infection	Whole Blood	0.212	500	57	GDS3919
111	Glioblastoma	Tumor Tissue	0.008	500	28	GDS1813
112	Sjogrens's Syndrome	Minor Salivary Gland	0.164	500	53	GDS3940
113	Ductal Carcinoma In Situ	Tumor Biopsy	0.036	500	34	GDS3853

Index	Condition	Tissue	Percentage Fibrotic	Network Size	miR-130/301 Rank	GEO ID
114	Hereditary Gingival Fibromatosis	Gingival Tissue	0.164	500	55	GDS1685
115	X-Linked Recessive Dystonia-Parkinsonism	Basal Ganglia	0.064	500	41	GDS1912
116	Hutchinson-Gilford Progeria Syndrome	Fibroblasts	0.068	500	43	GDS1504
117	Psoriasis	Lesional Skin	0.021	94	36	GDS3539
118	Astrocytic Tumor	Tumor Tissue	0.024	500	41	GDS1813
119	Ulcerative Colitis	Descending Colon, Inflamed	0.036	500	44	GDS3268
120	Anaplastic Oligoastrocytoma	Tumor Tissue	0.068	500	52	GDS1813
121	Oral Squamous Cell Carcinoma	Laser Capture Cell Isolates	0.184	500	70	GDS1584
122	Lethal Congenital Contracture Syndrome	Spinal Cord	0.064	500	52	GDS1295
123	Air Pollution Exposure, Adult	Peripheral Blood	0.128	500	63	GDS3325
124	Sickle Cell Disease	Platelets	0.039	204	50	GDS3318
125	Limb Immobilization	Skeletal Muscle	0.072	500	56	GDS2083
126	Rett Syndrome	Frontal Cortex	0.07	454	57	GDS2613

Index	Condition	Tissue	Percentage Fibrotic	Network Size	miR-130/301 Rank	GEO ID
127	Influenza Vaccination	Whole Blood	0.004	500	49	GDS3919
128	Obesity, Male	Cultured Abdominal Subcutaneous Mature Adipocytes	0.024	130	57	GDS1497
129	Emery-Dreifuss Muscular Dystrophy	Muscle Biopsy	0.008	500	58	GDS2855
130	Heat Stress	Skeletal Muscle	0.04	500	65	GDS4104
131	Chronic Lymphocytic Leukemia	Peripheral Blood B Cells	0.091	44	77	GDS4168
132	Weight Loss	Skeletal Muscle	0.024	500	69	GDS2089
133	Prostate Cancer	Stromal Tissue	0.091	22	92	GDS4114
134	Atopic Dermatitis	Non-Lesional Skin	0.012	486	86	GDS2382
135	H1N1 Infection	Peripheral Blood	0.091	22	113	GDS4240
136	West Nile Infection	Retinal Pigment Epithelium Cells	0.071	28	113	GDS4224
137	Barrett’s Esophagus	Endoscopic Biopsy	0.037	108	108	GDS4350

TABLE S2: Top 25 conserved miRNAs controlling the fibrosis network as ranked by one-way ANOVA score. Human conditions were grouped into cohorts based on their percentage overlap with the fibrosis signature. MiRNAs were ranked according to a one-way ANOVA means comparison test between their assigned spanning scores in each bin. High-scoring miRNAs were those carrying preferentially high spanning scores in diseases with a large fibrotic component, relative to their overall performance across diseases of all types.

Rank	ANOVA Score	ANOVA p-value	Family
1	25.499	1.2668E-12	miR-410/344de/344b-1-3p
2	12.140	1.4180E-05	miR-144
3	6.109	0.0007	miR-374ab
4	4.789	0.0098	miR-130ac/301ab/301b/301b-3p/454/721/4295/3666
5	4.539	0.0049	miR-155
6	3.790	0.0250	miR-27abc/27a-3p
7	3.508	0.0177	let-7/98/4458/4500
8	3.411	0.0201	miR-148ab-3p/152
9	3.366	0.0212	miR-19ab
10	3.293	0.0236	miR-128/128ab
11	3.186	0.0266	miR-590-3p
12	3.014	0.0331	miR-26ab/1297/4465
13	2.988	0.0342	miR-142-3p
14	2.672	0.0509	miR-101/101ab
15	2.659	0.0518	miR-300/381/539-3p
16	2.208	0.0911	miR-592/599
17	1.948	0.1466	miR-29abcd
18	1.832	0.1455	miR-145
19	1.702	0.1708	miR-17/17-5p/20ab/20b-5p/93/106ab/427/518a-3p/519d
20	1.691	0.1731	miR-22/22-3p

Rank	ANOVA Score	ANOVA p-value	Family
21	1.678	0.1758	miR-543
22	1.640	0.1843	miR-221/222/222ab/1928
23	1.632	0.1861	miR-199ab-5p
24	1.285	0.2835	miR-93/93a/105/106a/291a-3p/294/295/302abcde/372/373/428/519a/520be/520acd-3p/1378/1420ac
25	1.28404014883	0.2835	miR-205/205ab

TABLE S3: Networks connected with the fibrosis network and the miR-130/301 family share fibrotic genes with the PH network. The top 25% (n=34/137) networks are listed below, as ranked by overlap with the fibrosis network and miR-130/301 spanning score (see also **Fig.1B**). For each condition (column 4), we determined the fraction of disease genes shared with the previously described PH network [4]. In most cases, this fraction was dominated by fibrotic genes (*i.e.*, shared with the fibrosis network, column 5).

Index	Condition	Tissue	Network Fraction Shared with PH	Fibrotic Fraction of PH/Disease Overlap
1	Helicobacter Pylori Infection	Corpus Gastric Biopsy	0.138	0.891
2	Cerebral Palsy	Gracilis Muscle	0.050	0.560
3	Zaire Ebolavirus Infection	Macrophage	0.077	0.714
4	Alcoholic Hepatitis	Liver Tissue	0.053	0.717
5	Pterygium	Conjunctiva	0.075	0.653
6	Pituitary Gonadotrope	Tumor Tissue	0.062	0.710
7	Pituitary Null Cell Tumor	Tumor Tissue	0.058	0.724
8	Helicobacter Pylori Infection	Antumn Gastric Biopsy	0.073	0.466
9	Idiopathic Thrombocytopenic Purpura, Remission	CD3 Cells	0.054	0.815
10	Tuberous Sclerosis Complex Periungual Fibroma	Fibroblasts	0.060	0.600
11	Idiopathic Pulmonary Fibrosis	Whole Lung	0.056	0.589
12	Acute Rotavirus Infection	Peripheral Blood Mononuclear Cells	0.069	0.623
13	Familial Combined Hyperlidemia	Peripheral Blood	0.071	0.563
14	Autism Fragile X Type	Lymphoblastoid Cells	0.044	0.727
15	Schizophrenia	Postmortem Prefrontal Cortex	0.064	0.672

Index	Condition	Tissue	Network Fraction Shared with PH	Fibrotic Fraction of PH/Disease Overlap
16	Tuberous Sclerosis Complex Angiofibroma	Fibroblasts	0.072	0.556
17	Dermatomyositis	Skeletal Muscle Biopsy	0.064	0.594
18	ER Negative Breast Cancer	Tumor Tissue	0.070	0.457
19	Idiopathic Pulmonary Fibrosis	Fibroblasts	0.049	0.449
20	Bipolar Disorder	Postmortem Prefrontal Cortex	0.086	0.640
21	Head and Neck Squamous Carcinoma	Tumor Biopsy	0.067	0.597
22	ER Positive Breast Cancer	Tumor Tissue	0.076	0.526
23	UV Exposure	Skin Fibroblasts	0.058	0.621
24	Morbid Obesity	Omental Adipose Tissue	0.076	0.526
25	Idiopathic Thrombocytopenic Purpura, Active	CD3 Cells	0.074	0.459
26	Uterine Fibroid	Uterine Leiomyoma Tissue	0.076	0.579
27	Air Pollution Exposure, Child	Peripheral Blood	0.046	0.696
28	Scleroderma-Associated Pulmonary Fibrosis	Fibroblasts	0.048	0.563
29	Protein Deficiency	Skeletal Muscle	0.042	0.452
30	Aging	Frontal Cortex Male	0.068	0.618

Table S4: Clinical characteristics of patients suffering from liver fibrosis due to nonalcoholic fatty liver disease. NAFLD: Non-alcoholic fatty liver disease; NASH: Nonalcoholic steatohepatitis; **Grade steatosis**: **0**, <5%; **1**, 5-33%; **2**, 34-66%; **3**, >66%. **Lobular inflammation**: **0**, None; **1**, <2; **2**, 2-4; **3**, >4 per 200x**. Ballooning**: **0**, None; **1**, Few; **2**, Many. **Fibrosis:** 0; 1a; 1b; 1c; 2; 3; 4. **NAS (sum of scores for steatosis, lobular inflammation, and ballooning)**: **0-2**, (-); **3-4**, (+/-); **5-8**, (+).

Patient	Clinical description	Grade steatosis	Lobular inflammation	Ballooning	Fibrosis	NAS
1	Control	0	0	0	0	0
2	Control	0	0	0	0	0
3	Control	0	0	0	0	0
4	Control	0	0	0	0	0
5	NAFLD	1	1	0	0	2
6	NAFLD	1	1	0	0	2
7	NAFLD	1	1	0	0	2
8	NAFLD	1	0	1	0	2
9	NASH	3	1	1	0	5
10	NASH	2	2	1	0	5
11	NASH	2	2	1	0	5
12	NASH	2	1	2	1a	5
13	NASH	2	2	2	2	6
14	NASH	3	1	2	3	6
15	NASH	3	2	1	2	6
16	NASH	3	2	1	2	6

Supplemental References

1. Parikh, V. N., et al. MicroRNA-21 integrates pathogenic signaling to control pulmonary hypertension: Results of a network bioinformatics approach. *Circulation*. **125**,1520-1532 (2012).
2. Blondel, V. D., Guillaume, J. L., Hendrickx, J. M., de Kerchove, C. & Lambiotte, R. Local leaders in random networks. *Phys Rev E Stat Nonlin Soft Matter Phys*. **77**,036114 (2008).
3. Friedman, R. C., Farh, K. K., Burge, C. B. & Bartel, D. P. Most mammalian mrnas are conserved targets of microRNAs. *Genome Res*. **19**,92-105 (2009).
4. Bertero, T., et al. Systems-level regulation of microRNA networks by miR-130/301 promotes pulmonary hypertension. *J Clin Invest*. **124**,3514-3528 (2014).
5. Barry-Hamilton, V., et al. Allosteric inhibition of lysyl oxidase-like-2 impedes the development of a pathologic microenvironment. *Nat Med*. **16**,1009-1017 (2010).
6. Hu, B., et al. Multifocal epithelial tumors and field cancerization from loss of mesenchymal csl signaling. *Cell*. **149**,1207-1220 (2012).

Supplemental Figure Legends

Figure S1: A positive correlation exists between miR-130a expression, collagen crosslinking, and Yap1 activation in a mouse model of bleomycin-induced lung fibrosis and in lung fibrosis patients. A) In bleomycin-induced lung fibrosis in mice, expression levels of α–SMA, Pparγ, and Lrp8 were assessed by immunohistochemistry. Twenty-one days (n=10) after bleomycin injection, levels of Pparγ and Lrp8 were significantly decreased by bleomycin as compared with PBS (n=9). **B)** RT-qPCR confirmed an increase in collagen and the collagen cross-linking gene Lox in diseased lung. **C)** Serial lung sections derived from mice treated with bleomycin were stained for collagen (Picrosirius Red), miR-130a, and Yap1 **(see also Fig.2)**. In this panel (**C**), a higher magnification is presented. **D-E)** Serial lung sections from patients suffering from idiopathic pulmonary fibrosis were stained for collagen (Picrosirius Red), Yap1, and miR-130a (**D**). Quantification of miR-130a stain intensity and percentage of positively stained parenchymal cells revealed a correlation among miR-130a, Yap1 nuclear localization, and collagen crosslinking (**E**). Data are expressed as mean ± SEM (*P<0.05; ** P<0.01).

Figure S2: A positive correlation exists between miR-130a expression, collagen crosslinking, and Yap1 activation in a mouse model of CCl_4-induced liver fibrosis and in liver fibrosis patients. A) In CCl_4-induced liver fibrosis in mice, expression levels of α–SMA, Pparγ, and Lrp8 were assessed by immunohistochemistry. Four weeks (n=10) or six weeks (n=9) after CCl_4 exposure, levels of Pparγ and Lrp8 were

significantly decreased as compared with vehicle control (n=9, Oil). **B)** RT-qPCR confirmed an increase in collagen and the collagen cross-linking gene Lox in diseased liver. **C-D)** Serial hepatic sections derived from patients suffering from liver disease with varying levels of fibrosis were stained for YAP1 and miR-130a (**C**). Quantification of miR-130a stain intensity and percentage of YAP1 nuclear positive cells revealed a correlation among miR-130a and Yap1 nuclear localization (**D**). Data are expressed as mean ± SEM (*P<0.05; ** P<0.01).

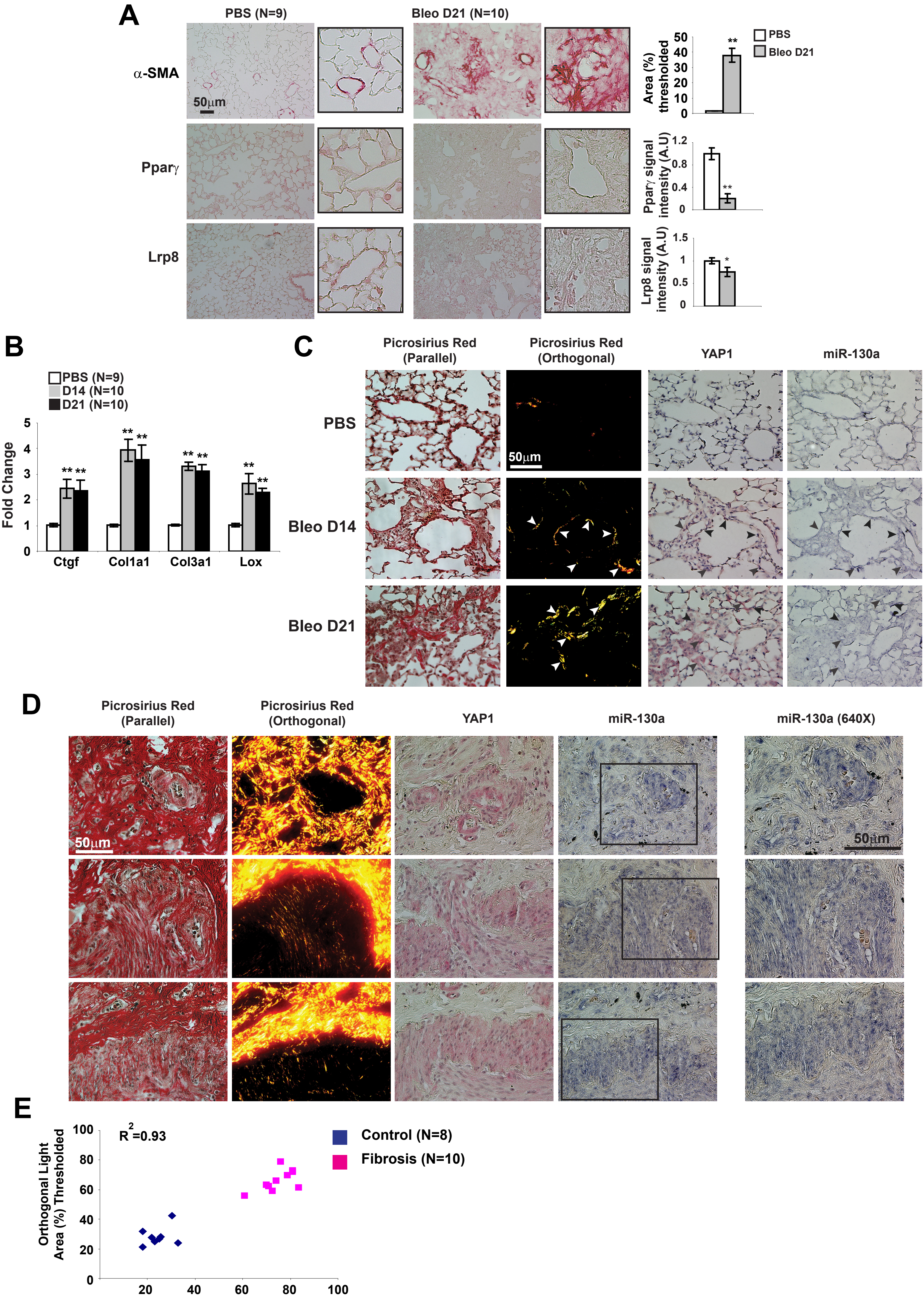

Bertero et al FigS1

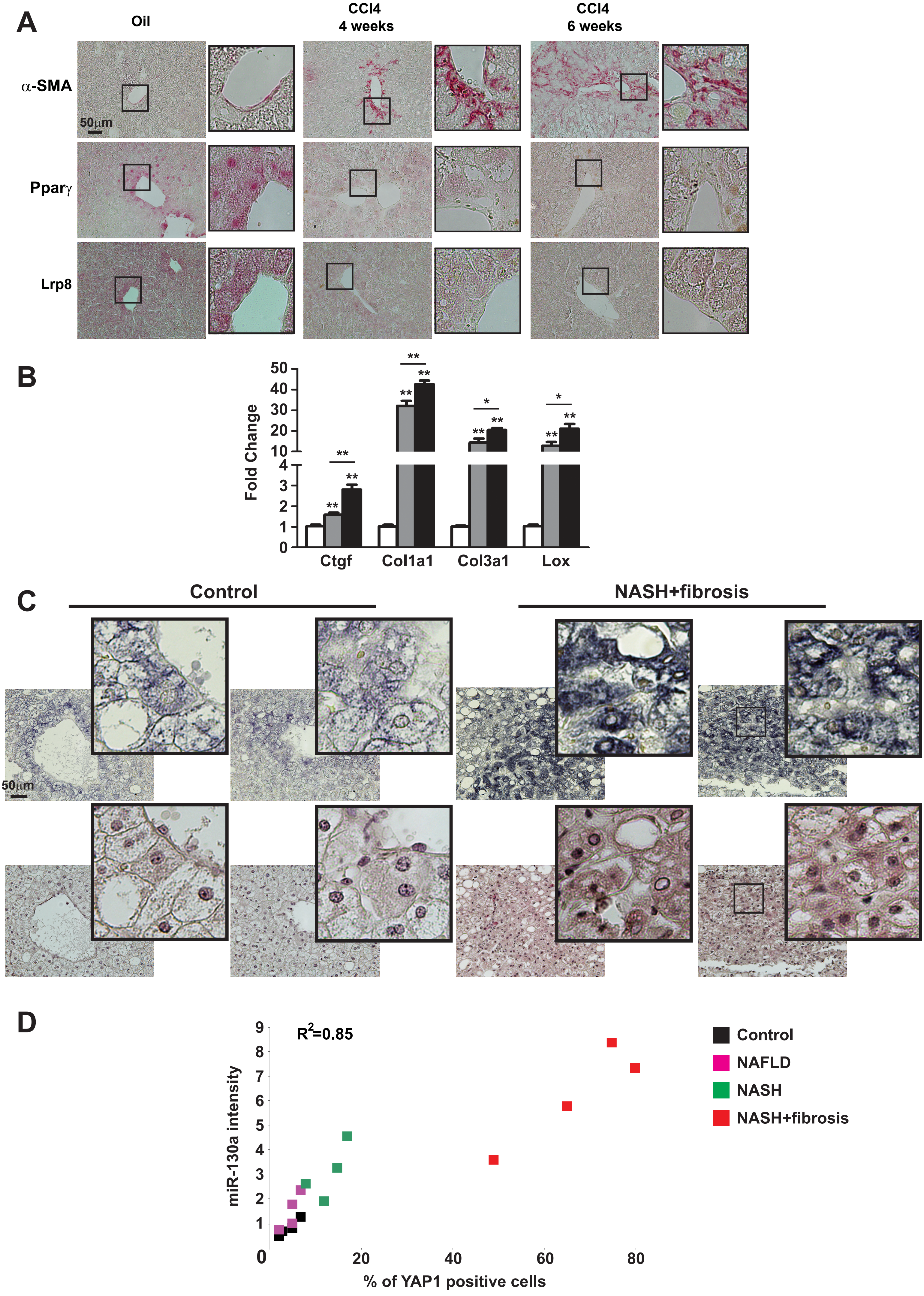

Bertero et al FigS2
